# Dexamethasone in Orthognathic Surgery: A Narrative Review of Pharmacological Rationale, Clinical Efficacy, Dosing Strategies, and Safety

**DOI:** 10.7759/cureus.118818

**Published:** 2026-10-30

**Authors:** Aljoury A Fatani, Rama Al-Qahtani, Dana Al-Wgait, Bader Fatani

**Affiliations:** 1 Pharmacy, Princess Nourah bint Abdulrahman University, Riyadh, SAU; 2 Dentistry, Princess Nourah bint Abdulrahman University, Riyadh, SAU; 3 Oral and Maxillofacial Surgery, King Saud University, Riyadh, SAU

**Keywords:** bilateral sagittal split osteotomy, corticosteroids, dexamethasone, le fort i osteotomy, narrative review, neurosensory disturbance, orthognathic surgery, postoperative edema, postoperative nausea and vomiting, postoperative pain

## Abstract

Dexamethasone is the corticosteroid most often given to patients undergoing orthognathic surgery, yet the dose, timing, and route that produce the best results are still debated. This narrative review brings together the pharmacological rationale for dexamethasone, the clinical evidence for its effects on facial edema, pain, postoperative nausea and vomiting (PONV), and neurosensory disturbance after orthognathic surgery, and the available data on dosing, timing, route of administration, and safety. Clinical trials in orthognathic patients were considered alongside systematic reviews and meta-analyses from orthognathic, oral and maxillofacial, facial plastic, and general surgery; trials confined to third molar and other minor dentoalveolar surgery were not included, and the evidence was synthesized narratively without statistical pooling. Perioperative corticosteroids consistently reduce early facial edema, with the clearest effect in the first 24 to 48 hours; most swelling resolves within weeks whether or not a steroid is given. Doses from 5 mg to 40 mg have been tested, and higher doses have not produced greater benefit. Lower pain scores, reduced opioid consumption, and a lower incidence of PONV are supported mainly by large meta-analyses in general surgical populations, with modest effect sizes. Systemic dexamethasone has not been shown to hasten recovery of inferior alveolar nerve function, and topical application to the exposed nerve has given conflicting results. A single preoperative intravenous dose is the best-supported regimen. Continuing dexamethasone after surgery has rarely been tested on its own, and the one orthognathic trial with a separate postoperative arm found no added benefit. Short perioperative courses appear safe, the main measurable effect being a transient rise in blood glucose. A single moderate preoperative dose of dexamethasone is therefore a reasonable standard, and routine postoperative dosing cannot be recommended until trials that isolate its contribution become available.

## Introduction and background

Orthognathic surgery corrects dentofacial deformities that orthodontic treatment alone cannot resolve, with the aim of improving function, facial esthetics, and psychosocial well-being. Although surgical technique and perioperative care have advanced considerably, edema, pain, trismus, and neurosensory disturbance remain common after these procedures and can slow recovery and reduce patient satisfaction [1,2]. Edema and sensory deficit deserve particular attention because they interfere with eating, speech, and oral hygiene during the healing period and lower quality of life for as long as they last [3,4].

Postoperative edema follows the wide soft-tissue dissection, periosteal stripping, and osteotomies that the surgery requires. These steps increase vascular permeability and allow inflammatory exudate to collect in the tissues of the face [5,6]. Swelling usually peaks 48 to 72 hours after surgery and then subsides over the following weeks [3,7]. Neurosensory disturbance is seen most often after mandibular procedures, particularly bilateral sagittal split osteotomy (BSSO), because the inferior alveolar nerve lies within the operative field and can be stretched, compressed, or cut [1,2]. The resulting paresthesia, hypoesthesia, or anesthesia of the lower lip and chin is temporary in most patients but persists in some [1].

Corticosteroids have been used for decades to blunt the inflammatory response to oral and maxillofacial surgery [5]. Dexamethasone is the agent most often chosen because of its potency, long duration of action, and lack of mineralocorticoid effect [5,6]. By inhibiting phospholipase A2 it limits the release of arachidonic acid and, in turn, the production of the prostaglandins and leukotrienes that drive vasodilatation and capillary leakage [5]. In the wider surgical literature, a single perioperative dose also lowers pain scores and opioid consumption and reduces the incidence of postoperative nausea and vomiting (PONV) [8-10].

Several trials have examined dexamethasone specifically in orthognathic patients. Weber and Griffin reported in 1994 that preoperative intravenous dexamethasone reduced facial edema and the C-reactive protein response after BSSO [3]. More recently, Semper-Hogg et al. used three-dimensional surface scanning to show less swelling after a single preoperative dose, although nerve function did not recover any faster [11]. Other trials have compared doses [7,12], tested betamethasone [13], or applied dexamethasone directly onto the exposed inferior alveolar nerve, with conflicting results [14,15]. Questions therefore remain about the most effective dose, the value of continuing treatment after surgery, and whether local delivery offers anything beyond the intravenous route [6,7].

Systematic reviews and meta-analyses broadly support corticosteroids for reducing edema after orthognathic surgery [4,16-18], but differences in study design and treatment protocols have prevented consensus on a regimen. This narrative review summarizes the pharmacological rationale for dexamethasone, the clinical evidence for its effects on edema, pain, PONV, and neurosensory disturbance after orthognathic surgery, and what is known about dose, timing, route of administration, and safety. Particular attention is paid to whether continuing dexamethasone into the postoperative period adds anything to a single preoperative dose. Publications were identified through searches of PubMed/MEDLINE and Google Scholar using combinations of the terms "dexamethasone", "corticosteroid", "glucocorticoid", "orthognathic surgery", "sagittal split osteotomy", "Le Fort I", "edema", "pain", "nausea", and "neurosensory", supplemented by hand-searching the reference lists of the articles retrieved.

Clinical trials in orthognathic patients were given priority; systematic reviews and meta-analyses from oral and maxillofacial, facial plastic, and general surgery were drawn on where orthognathic data were lacking. Trials of dexamethasone in third molar and other minor dentoalveolar surgery were not included, because the extent of soft-tissue dissection, the duration of surgery, and the pattern of postoperative morbidity in these procedures differ substantially from those of orthognathic surgery, so their results cannot be assumed to apply.

Twenty-six publications were included. Because this is a narrative review, no meta-analysis, meta-regression, or other quantitative pooling was undertaken; the effect estimates, 95% confidence intervals (CIs), and p-values quoted in the text are those reported by the original authors and were not re-analyzed.

## Review

Orthognathic surgery and its postoperative sequelae

Orthognathic surgery comprises the osteotomies used to correct skeletal jaw discrepancies that orthodontics alone cannot resolve. Patients typically present with a skeletal Class II or Class III relationship, an anterior open bite, or facial asymmetry, and many report difficulty with chewing, speech, or breathing as well as concern about their appearance [1,2]. The maxilla is repositioned through a Le Fort I osteotomy, which allows movement in the vertical, sagittal, and transverse planes, and the mandible through BSSO, which permits advancement or setback while preserving broad bony contact for healing and rigid fixation. A genioplasty may be added when the chin needs separate correction, and bimaxillary surgery combines maxillary and mandibular osteotomies in a single operation [1,2]. Treatment is preceded by a period of orthodontic decompensation, usually lasting one to two years, and by cephalometric or, increasingly, three-dimensional virtual planning of the skeletal movements.

Some facial swelling is unavoidable. The wide subperiosteal dissection and bone cuts disrupt local vessels and provoke an inflammatory response that raises capillary permeability and drives fluid into the interstitial tissues of the face [5,6]. Swelling reaches its maximum within two to three days and then subsides over several weeks, although some residual fullness can persist for months [3,7]. Bimaxillary procedures produce more swelling than single-jaw surgery [11]. Marked swelling restricts mouth opening, makes a soft diet and oral hygiene harder to manage, and interferes with speech, and colors the patient's perception of the whole experience [3,4].

Altered sensation in the territory of the trigeminal nerve is the most consistently reported sequela of orthognathic surgery, and the risk is greatest with mandibular procedures. During BSSO, the inferior alveolar nerve lies within the osteotomy site, where it can be injured by the bone cut itself, by traction as the segments are separated, by instrument pressure or frictional heat, or by compromise of its blood supply [1,2]. The resulting sensory change ranges from a slight reduction in touch perception to complete anesthesia of the lower lip, chin, and labial mucosa [1]. Almost all patients notice some alteration immediately after BSSO; in most it resolves within the first year, but a small proportion, about 3% in one series, are left with persistent paresthesia [1]. In a Finnish series of 655 patients, the 574 who underwent a mandibular osteotomy had a mild sensory deficit in 32% of cases and a deficit that 3% of patients found disturbing [2]. Maxillary osteotomies carry a lower risk, but subjective sensory change in the infraorbital territory has been reported in 7% to 60% of patients one year after Le Fort I osteotomy, depending on the site tested [1]. Persistent numbness affects eating, speaking, and grooming, and it is one of the complications patients ask about most before surgery [1,2].

Pharmacology of dexamethasone

Dexamethasone is a synthetic fluorinated glucocorticoid with roughly 25 to 30 times the anti-inflammatory potency of hydrocortisone, a biological half-life of 36 to 72 hours, and negligible mineralocorticoid activity [5]. Glucocorticoids act through cytoplasmic receptors to alter the transcription of genes that govern inflammation. They induce lipocortin, which inhibits phospholipase A2 and so reduces the release of arachidonic acid, the common precursor of prostaglandins and leukotrienes [5,6]. They also suppress the production of pro-inflammatory cytokines such as interleukin-1 and tumor necrosis factor, reduce the migration of leukocytes into injured tissue, lower capillary permeability, and stabilize lysosomal membranes [5,6]. The net effect in the operative field is less exudate and less swelling, and because dexamethasone acts for two to three days, a single preoperative dose spans the period in which edema would otherwise peak [6].

The same actions account for the drug's adverse effects, which depend largely on dose and duration. Prolonged therapy causes adrenal suppression, hyperglycemia, osteoporosis, impaired wound healing, susceptibility to infection, and psychiatric symptoms ranging from insomnia and mood change to, rarely, frank psychosis [5,6,16]. Short perioperative courses carry a much smaller risk. A transient rise in blood glucose is measurable even after a single dose and matters most in patients with diabetes [8], whereas the feared effects on wound healing and infection have not been borne out in oral surgery trials [5,16]. Hypersensitivity reactions are rare. Avascular osteonecrosis and steroid-induced psychosis have been reported with the higher doses used in orthognathic surgery, although they remain exceptional [16].

Clinical evidence in orthognathic surgery

The outcomes most often studied are facial edema, pain, PONV, and neurosensory disturbance of the inferior alveolar nerve, which are also the problems patients most frequently report after surgery [1,2]. Eight clinical trials in orthognathic patients have evaluated dexamethasone or a comparable corticosteroid; their designs and main findings are summarized in Table 1 [3,7,11-15,19].

**Table 1:** Clinical trials of corticosteroids in orthognathic surgery 3D: three-dimensional; BSSO: bilateral sagittal split osteotomy; CRP: C-reactive protein; CT: computed tomography; ESR: erythrocyte sedimentation rate; h: hours; IV: intravenous; min: minutes; PONV: postoperative nausea and vomiting; RCT: randomized controlled trial; TOVRO: transoral vertical ramus osteotomy. Percentages and p-values are those reported by the original authors; no re-analysis was performed.

Study	Design and sample size	Procedure	Corticosteroid and regimen	Outcome measures	Main finding
Schaberg et al., 1984 [19]	Clinical trial, n = 39	Le Fort I osteotomy; TOVRO	Methylprednisolone IV	CT scans before surgery and at 24 and 72 h	Edema reduced by 61% at 24 h and 10% at 72 h after Le Fort I osteotomy, and by 38% at 24 h and 45% at 72 h after TOVRO
Weber and Griffin, 1994 [3]	Double-blind RCT, n = 23	BSSO	Dexamethasone 16 mg IV before surgery, with or without three 8 mg doses every 6 h after surgery, versus placebo	Standardized photographs; CRP, ESR, blood counts	Edema lower on day 1 only; CRP lower on days 1-3 in both dexamethasone groups; no difference between the two regimens
Pourdanesh et al., 2014 [14]	Split-mouth RCT, n = 18 (36 sides)	BSSO	Dexamethasone 4 mg/mL poured onto the exposed inferior alveolar nerve during the split and again before fixation	Light touch, direction of movement, two-point discrimination, thermal and pin-prick testing	No difference between treated and control sides at any interval
Widar et al., 2015 [13]	RCT, n = 37	BSSO	Betamethasone 16 mg IV as a single preoperative dose (n = 11), or 4 mg orally the day before surgery, 8 mg IV immediately before surgery, and 4 mg orally the day after (n = 14), versus no steroid (n = 12)	Facial edema, pain, and lip and chin sensation at 1 day, 7 days, 2 months, and 6 months	Less edema on day 1 in both betamethasone groups (p = 0.017); no significant difference in pain; no reduction in neurosensory disturbance
Semper-Hogg et al., 2017 [11]	RCT, n = 38	Le Fort I osteotomy; BSSO; bimaxillary surgery	Dexamethasone 40 mg IV before surgery versus no steroid	3D optical surface scans to day 90; thermal testing of the inferior alveolar and infraorbital nerves	Less swelling after Le Fort I osteotomy (−11%, p = 0.048), BSSO (−18%, p = 0.046), and bimaxillary surgery (−39%, p < 0.001); no effect on inferior alveolar (p = 0.746) or infraorbital (p = 0.465) nerve function
Abukawa et al., 2017 [12]	Double-blind RCT, n = 24	BSSO	Dexamethasone 16 mg versus 8 mg versus none, IV before surgery	Masseter and cheek thickness; maximum incisal opening; lip and chin sensation; blood tests on days 1-3	16 mg limited the increase in masseter thickness at 24 h (38.4% versus 57.7% with 8 mg and 56.1% in controls; p < 0.05 for 16 mg versus control); no difference in mouth opening or sensation
Lin et al., 2017 [7]	Double-blind RCT, n = 68 enrolled (56 analyzed)	Orthognathic surgery	Dexamethasone 5 mg versus 15 mg	3D photogrammetry at 48 h, 1 week, 1 month, and 6 months; PONV	No difference between doses in swelling at any time point or in PONV; swelling 86% resolved at 1 month
Logvynenko et al., 2025 [15]	Parallel-group RCT, n = 22 (11 per group; 44 osteotomy sides)	BSSO	Dexamethasone 4 mg/mL on gauze applied to the exposed nerve for 10 min; all patients received postoperative dexamethasone 12 mg tapering to 4 mg	Light-touch mapping of the numb area on the lip and chin to 6 months	Smaller areas of numbness in the dexamethasone group from 1 week to 3 months (p < 0.001 in all four lip and chin segments at 3 months); groups similar at 6 months

They are supplemented by systematic reviews confined to orthognathic surgery [4,16-18,20] and by meta-analyses from general and facial surgery [8,9,21,22].

Facial Edema

Facial edema is the outcome most consistently improved by corticosteroids. The earliest controlled evidence came from Schaberg et al., who measured swelling on CT scans in 39 patients given intravenous methylprednisolone [19]. Edema was 61% lower at 24 hours after Le Fort I osteotomy, although the difference had shrunk to 10% by 72 hours; after transoral vertical ramus osteotomy the reductions were 38% at 24 hours and 45% at 72 hours [19]. Weber and Griffin then conducted an early double-blind, placebo-controlled trial of dexamethasone in 23 patients undergoing BSSO [3]. Patients received placebo, 16 mg of intravenous dexamethasone before surgery, or the same preoperative dose followed by three 8 mg doses at six-hour intervals. Edema, measured by computer analysis of standardized photographs, was significantly lower with dexamethasone on the first postoperative day but not thereafter, and the two dexamethasone regimens did not differ from each other [3].

More recent trials have relied on objective three-dimensional measurement. Semper-Hogg et al. randomized 38 patients to a single 40 mg intravenous dose before surgery or no steroid and quantified swelling with optical surface scans [11]. Swelling was significantly lower in the dexamethasone group after Le Fort I osteotomy (11% less on day 1, p=0.048), BSSO (18% less on day two, p=0.046), and bimaxillary surgery (39% less on day two, p<0.001), the last showing the largest reduction [11]. Abukawa et al. compared 16 mg, 8 mg, and no dexamethasone in 24 patients undergoing BSSO [12]. At 24 hours the masseter had thickened by 38.4% with 16 mg, 57.7% with 8 mg, and 56.1% without steroid; only the 16 mg dose differed significantly from control (p<0.05, eight patients per group) [12]. Betamethasone behaves in much the same way. Widar et al. found that a single 16 mg dose or divided doses of 4, 8, and 4 mg reduced facial swelling on day one after BSSO compared with no steroid (p=0.017), with no difference between the two regimens [13]. Lin et al. randomized 68 patients to 5 mg or 15 mg of dexamethasone and followed swelling by three dimensional photogrammetry for six months [7]. Swelling peaked at about 48 hours, had subsided by an average of 86% at one month, and did not differ between the doses at any time point [7].

Systematic reviews reach the same conclusion. De Lima et al. included eight studies with 315 patients, none of which provided data suitable for pooling, and concluded that corticosteroids improve the regression of facial edema regardless of dose, with no notable edema remaining after four months [17]. Jean et al. identified eight randomized trials with 234 participants and, pooling the three trials that could be combined (80 patients), found a standardized mean difference (SMD) of −1.07 (95% CI −1.99 to −0.16) in favor of corticosteroids for facial edema, although every trial was judged to be at unclear risk of bias [18]. Dan et al. found that intravenous doses equivalent to at least 85 mg of methylprednisolone were sufficient to reduce edema after orthognathic surgery [16], a conclusion echoed in an evidence-based commentary on their review [23]. Galdino-Santos et al., in a systematic review of 18 randomized trials of perioperative therapies, eight of which (n=349) could be pooled, listed dexamethasone and betamethasone among the treatments that appear to reduce edema, although a dexamethasone-specific pooled estimate could not be derived [4].

Pain and Postoperative Nausea and Vomiting (PONV)

Pain has seldom been the primary outcome of orthognathic dexamethasone trials, so most of the evidence comes from broader surgical populations. Of the trials in Table 1, only Widar et al. recorded pain, and they found no significant difference between betamethasone and control [13]. Waldron et al. pooled 45 trials with 5,796 patients in which a single perioperative dose of dexamethasone (1.25 to 20 mg) was compared with placebo [8]. Pain scores were lower at two hours (mean difference −0.49, 95% CI −0.83 to −0.15) and at 24 hours (mean difference −0.48, 95% CI −0.62 to −0.35), opioid consumption at 24 hours was reduced (mean difference −2.33 mg of morphine equivalents, 95% CI −4.39 to −0.26), fewer patients needed rescue analgesia (relative risk 0.80, 95% CI 0.69 to 0.93), and time in the recovery unit was about five minutes shorter (mean difference −5.32 minutes, 95% CI −10.49 to −0.15) [8]. No dose-response relationship was found for the opioid-sparing effect [8]. De Oliveira et al. analyzed 24 trials with 2,751 patients [9]. Dexamethasone reduced pain both at rest and on movement at 24 hours; intermediate (0.11 to 0.2 mg/kg) and high (0.21 mg/kg or more) doses reduced opioid consumption, whereas low doses (less than 0.1 mg/kg) did not, and preoperative administration produced a more consistent analgesic effect than intraoperative administration [9].

In oral and maxillofacial surgery more broadly, Rahpeyma et al. reviewed nine randomized trials of pharmacological and non-pharmacological pain control and found that preoperative ibuprofen markedly reduced pain, and that dexamethasone, oxycodone, and laser therapy were effective in relieving postoperative discomfort [24]. Alyahya et al., in a systematic review of randomized trials in orthognathic patients, found that pre-emptive analgesia (SMD −1.33, 95% CI −2.03 to −0.63, p=0.001) and low-level laser therapy (SMD −0.69, 95% CI −1.17 to −0.21, p=0.005) reduced pain in the first 48 hours, although they rated the evidence as very low quality [20].

The antiemetic effect of dexamethasone is well established. In a placebo-controlled trial of 60 women undergoing major gynecological surgery, 10 mg of intravenous dexamethasone at induction reduced the incidence of vomiting in the first 24 hours from 63.3% to 20.0% (p<0.01), with little effect on pain [10]. In orthognathic patients, Lin et al. observed similar rates of PONV with 5 mg and 15 mg, which suggests that a low dose is enough for this purpose [7]. Chegini and Dhariwal identified PONV, together with swelling and pain, as a principal reason for giving steroids in orthognathic surgery [6].

Neurosensory Disturbance

Inferior alveolar nerve disturbance is the most common complication of mandibular osteotomy [1,2]. Corticosteroids have been proposed to reduce perineural edema and so limit secondary compression of the nerve, and Dan et al. noted that several trials hinted at a neuroregenerative effect, although the data did not permit statistical analysis [16]. The randomized trials of systemic administration have been uniformly negative. Semper-Hogg et al. found no effect of 40 mg of dexamethasone on inferior alveolar (p=0.746) or infraorbital (p=0.465) nerve function over 90 days [11]. Betamethasone did not reduce neurosensory disturbance over six months after BSSO [13], and lip and chin sensation did not differ between dexamethasone doses in the trial by Abukawa et al. [12]. De Lima et al. concluded that corticosteroids did not influence neurosensory disorders, which resolved within about three months with or without them [17].

Two trials applied dexamethasone directly to the exposed nerve during BSSO and reached different conclusions. Pourdanesh et al. used a split-mouth design in 18 patients, pouring a 4 mg/mL solution onto the distal segment of the nerve during the split and again before fixation [14]. Light touch, direction of movement, two-point discrimination, thermal, and pin-prick testing showed no difference between the two sides at any interval, and the authors advised against the technique [14]. Logvynenko et al. randomized 22 patients (11 per group, 44 osteotomy sides) to the application of gauze soaked in 4 mg/mL dexamethasone to the exposed nerve for 10 minutes before osteosynthesis or to conventional BSSO [15]. Every patient also received a tapering postoperative course of systemic dexamethasone that began at 12 mg on the first day and fell to 4 mg. The area of numbness on the lip and chin, mapped in four segments, was smaller in the dexamethasone group in one segment at one week (p=0.028), in two segments at one month (p=0.013 and p=0.002), and in all four segments at three months (p<0.001), but the groups were indistinguishable by six months [15]. Because both groups received systemic dexamethasone, the trial tested topical application on top of systemic treatment rather than instead of it.

Inflammatory Markers and Jaw Function

Dexamethasone measurably dampens the systemic inflammatory response to orthognathic surgery. Weber and Griffin found that C-reactive protein was significantly lower on postoperative days one to three in both dexamethasone groups than with placebo, and they regarded it as the most sensitive index of the drug's effect [3]. Abukawa et al. observed that lymphocyte counts remained at preoperative levels in patients given 16 mg or 8 mg but fell in controls [12]. Functional benefit is harder to demonstrate. Abukawa et al. found no difference in maximum incisal opening between 16 mg, 8 mg, and no dexamethasone [12], and theirs is the only trial in Table 1 to have measured it. Mouth opening after orthognathic surgery is restricted by occlusal splints, guiding elastics, and the osteotomies themselves as much as by inflammatory swelling, so any effect of dexamethasone on jaw function in this population remains unproven.

Dose, timing, and route of administration

Dexamethasone doses in the orthognathic trials ranged from 5 mg to 40 mg (Table 1). Lin et al. found 5 mg and 15 mg equally effective for swelling and PONV [7], whereas Abukawa et al. found 16 mg effective and 8 mg not [12]. De Lima et al. judged the anti-edema effect to be independent of dose [17], and Dan et al. placed the threshold at about 85 mg of methylprednisolone equivalent, which corresponds to roughly 16 mg of dexamethasone [16]. In the wider surgical literature, analgesic benefit required more than 0.1 mg/kg, and higher doses added nothing [8,9]. Taken together, these data point to a ceiling effect: a moderate dose appears sufficient, and very high doses lack evidence of further benefit.

Most orthognathic trials gave the drug before surgery or at induction [3,11,12], and Chegini and Dhariwal concluded that preoperative administration is supported by the evidence while the value of continuing treatment after surgery remains open [6]. Only one orthognathic trial has included a separate postoperative arm. In Weber and Griffin's study, the group that received three further 8 mg doses in the 18 hours after surgery fared no better than the group given a single preoperative dose, and the benefit of either regimen was confined to the first postoperative day [3]. The divided betamethasone regimen used by Widar et al., which included a 4 mg oral dose on the first postoperative day, was no more effective than a single preoperative dose of 16 mg [13], and in the trial by Logvynenko et al. both groups received postoperative dexamethasone, so its independent contribution could not be assessed [15]. In general surgery, preoperative dosing gave more consistent analgesia than intraoperative dosing [9]. The one body of evidence favoring combined pre- and postoperative administration comes from facial plastic surgery. Aldhabaan et al. pooled 10 of 19 eligible studies (439 patients) and found that perioperative corticosteroids reduced periorbital edema (SMD −0.82, 95% CI −1.37 to −0.26) and ecchymosis (SMD −0.95, 95% CI −1.32 to −0.57) on the first postoperative day, that the difference was no longer significant at days three and seven, and that adding postoperative doses to a preoperative dose was associated with less edema and ecchymosis than preoperative dosing alone, a comparison for which no pooled estimate is given in the main report [21]. Their analysis covered facial plastic and reconstructive procedures such as rhinoplasty, in which dexamethasone has clear short-term benefits [22], and these operations differ considerably from orthognathic surgery in the extent of bone and soft-tissue trauma. The pharmacology offers part of the explanation for this pattern: with a biological half-life of 36 to 72 hours, a single preoperative dose already covers the period in which edema would otherwise peak [5,6].

Intravenous administration is standard in orthognathic surgery [3,11,12]. Topical application to the inferior alveolar nerve is the only local route that has been tested in orthognathic patients, and the two trials disagree [14,15]. Intramuscular, oral, and submucosal administration have been used in oral surgery [5,16], but none of these routes has been compared with intravenous dosing in orthognathic patients, and whether a local route would allow a lower total dose is unknown. Practice reflects this uncertainty: there is no nationally agreed steroid regimen for orthognathic surgery in the United Kingdom [6], and in a Finnish survey every consultant oral and maxillofacial surgeon who responded gave glucocorticoids for orthognathic surgery, with regimens ranging from a single 5 mg dose to a total of 116 mg of dexamethasone equivalent [25].

Other corticosteroids and non-steroidal alternatives

Dexamethasone is not the only corticosteroid that has been studied in orthognathic surgery. Methylprednisolone [19] and betamethasone [13] produced similar early reductions in edema, and when Dan et al. converted all regimens to methylprednisolone equivalents, no single agent emerged as clearly superior [16]. Non-steroidal measures have also been evaluated. Galdino-Santos et al. found moderate-certainty evidence that thermotherapy reduces edema at 48 hours and 30 days and high-certainty evidence that manual lymphatic drainage and laser therapy do not, while enzyme therapy, Venoplant, and kinesiology taping appeared to help but with less certain evidence [4]. For pain and PONV, preoperative dexmedetomidine, an alpha-2 adrenergic agonist rather than a corticosteroid, lowered pain scores at every time point in the first 24 hours and reduced the incidence of nausea from 46.7% to 3.3% in a triple-blind randomized trial of 60 patients undergoing bimaxillary surgery [26]. These options may complement dexamethasone or suit patients in whom steroids are relatively contraindicated. Evidence from other surgical fields supports the anti-inflammatory effect of dexamethasone: in rhinoplasty, a meta-analysis of 10 studies with 374 participants found medium-to-large reductions in edema (Hedges' g −1.09), ecchymosis (−1.03), and intraoperative blood loss (−0.69) [22].

Safety

Short perioperative courses of corticosteroids appear safe. Gersema and Baker reviewed oral surgery trials using 80 to 625 mg of hydrocortisone equivalent and found no significant adverse reactions [5]. Dan et al. found no increase in the risk of infection (relative risk 1.0041, p=0.89) and no reports of impaired healing [16], and Waldron et al. likewise found no increase in wound infection or delayed healing, although blood glucose was 0.39 mmol/L higher at 24 hours (95% CI 0.04 to 0.74) [8]. Theoretical risks nevertheless remain. They include adrenal suppression and impaired wound healing with longer courses [5], and rare events such as avascular osteonecrosis and steroid-induced psychosis, which Dan et al. flagged particularly for the higher doses used in orthognathic surgery [16]. The rise in blood glucose matters most in patients with diabetes [8]. Jean et al. observed that adverse events were inconsistently screened for and reported in the orthognathic trials; where they were recorded, they included surgical-site bleeding, hypersensitivity, and stomach discomfort [18]. These risks grow with the length of the course, which strengthens the case for a clear demonstration of benefit before routine postoperative dosing is adopted. In practice, corticosteroids remain in widespread but unstandardized use, and the low cost and minimal side effects of current regimens [6] go some way to explaining why.

Where the evidence leaves clinicians

Several conclusions can be drawn from the trials summarized above. The anti-edema effect of perioperative corticosteroids is real but front-loaded: it is largest in the first 24 to 48 hours and has usually disappeared by the end of the first week, and most swelling resolves within weeks whether or not a steroid is given [3,7,11-13,17,19]. Because dexamethasone is long-acting, a single preoperative dose already covers the early inflammatory peak, which may be why added postoperative doses have not shown an extra benefit [3,6]. A moderate dose is enough; doses of 5 mg to 15 mg were equivalent in the largest trial [7], 16 mg outperformed 8 mg in another [12], and nothing suggests that 40 mg achieves more than 16 mg [11,17].

The benefits for pain and PONV are statistically robust but modest, and they are borrowed almost entirely from other surgical populations [8-10]; the one orthognathic trial that recorded pain found no difference [13]. Dexamethasone is therefore best regarded as one component of multimodal analgesia and antiemetic prophylaxis rather than as the main analgesic [8,9,20]. For neurosensory disturbance, the systemic trials are consistently negative [11-13,17]. This is biologically plausible: much of the nerve injury during sagittal split osteotomy is mechanical, whereas corticosteroids act mainly on the inflammatory and edematous component around the nerve, so any benefit is likely to be limited and temporary. The earlier recovery reported with topical application [15] is intriguing but rests on a single small trial that contradicts an earlier split-mouth study [14], and it needs replication before it can be recommended.

Limitations of the evidence and of this review

The evidence base has some weaknesses. The orthognathic trials are small, ranging from 18 to 68 patients [3,7,11-15], and they differ in corticosteroid, dose, timing, surgical procedure, and outcome measurement. Swelling has been assessed with standardized photographs [3], CT [19], soft-tissue thickness [12], three-dimensional surface scans [11], and photogrammetry [7], which limits direct comparison and has prevented meta-analyses from pooling dexamethasone-specific estimates [4]. Every trial in the most recent meta-analysis was judged to be at unclear risk of bias [18]. Most importantly for the question posed here, postoperative dexamethasone has rarely been tested in isolation: nearly all trials gave the drug before or during surgery, so the specific contribution of postoperative dosing remains unclear [3,6]. Evidence on pain and PONV is largely extrapolated from general surgery [8-10] and from the wider oral and maxillofacial literature [24]. This review has limitations of its own. As a narrative review it did not use a formal systematic search, duplicate study selection, risk-of-bias assessment, or quantitative synthesis, so study selection may be subject to bias, and its conclusions should be read as a qualitative summary of the available evidence. No statistical analyses were performed by the authors: the effect sizes, CIs, and p-values cited are those of the original publications, were derived with different outcome measures and scales, and should not be compared directly across studies. Finally, trials from third molar and other minor oral surgery were deliberately excluded to keep the review specific to orthognathic patients, so some evidence on alternative routes of administration and on jaw function was not considered.

## Conclusions

Perioperative dexamethasone reduces facial edema after orthognathic surgery, but the effect is concentrated in the first one to two days, and most swelling resolves within weeks whether or not a steroid is given. A single preoperative intravenous dose of moderate size is the best-supported regimen: higher doses have not produced greater benefit, and the only orthognathic trial to add postoperative doses to a preoperative dose found nothing extra. Lower pain scores, less opioid use, and less postoperative nausea and vomiting are reasonable additional expectations, although the evidence for them comes mainly from other surgical populations and the effects are modest. Systemic dexamethasone does not hasten recovery of inferior alveolar nerve function, and the early benefit reported with topical application to the nerve awaits confirmation.

Short perioperative courses appear safe, with a transient rise in blood glucose as the main measurable effect, but the risks of adrenal suppression, impaired healing, and infection increase with the length of treatment. Until trials that isolate the postoperative period are available, routine continuation of dexamethasone after orthognathic surgery cannot be recommended on current evidence, and a single moderate preoperative dose remains a reasonable standard of care. Adequately powered trials comparing a single preoperative dose with a preoperative dose plus a short postoperative course, using objective three-dimensional measurement of swelling and standardized neurosensory testing, would settle the question.
